# TMDB: A literature-curated database for small molecular compounds found from tea

**DOI:** 10.1186/s12870-014-0243-1

**Published:** 2014-09-16

**Authors:** Yi Yue, Gang-Xiu Chu, Xue-Shi Liu, Xing Tang, Wei Wang, Guang-Jin Liu, Tao Yang, Tie-Jun Ling, Xiao-Gang Wang, Zheng-Zhu Zhang, Tao Xia, Xiao-Chun Wan, Guan-Hu Bao

**Affiliations:** Key Laboratory of Tea Biochemistry and Biotechnology, Anhui Agricultural University, 130 West Changjiang Road, Hefei, Anhui Province 230036 China

**Keywords:** Tea, *Camellia sinensis*, Small molecular, Compounds, Metabolites

## Abstract

**Background:**

Tea is one of the most consumed beverages worldwide. The healthy effects of tea are attributed to a wealthy of different chemical components from tea. Thousands of studies on the chemical constituents of tea had been reported. However, data from these individual reports have not been collected into a single database. The lack of a curated database of related information limits research in this field, and thus a cohesive database system should necessarily be constructed for data deposit and further application.

**Description:**

The Tea Metabolome database (TMDB), a manually curated and web-accessible database, was developed to provide detailed, searchable descriptions of small molecular compounds found in *Camellia spp.* esp. in the plant *Camellia sinensis* and compounds in its manufactured products (different kinds of tea infusion). TMDB is currently the most complete and comprehensive curated collection of tea compounds data in the world. It contains records for more than 1393 constituents found in tea with information gathered from 364 published books, journal articles, and electronic databases. It also contains experimental ^1^H NMR and ^13^C NMR data collected from the purified reference compounds or collected from other database resources such as HMDB. TMDB interface allows users to retrieve tea compounds entries by keyword search using compound name, formula, occurrence, and CAS register number. Each entry in the TMDB contains an average of 24 separate data fields including its original plant species, compound structure, formula, molecular weight, name, CAS registry number, compound types, compound uses including healthy benefits, reference literatures, NMR, MS data, and the corresponding ID from databases such as HMDB and Pubmed. Users can also contribute novel regulatory entries by using a web-based submission page. The TMDB database is freely accessible from the URL of http://pcsb.ahau.edu.cn:8080/TCDB/index.jsp. The TMDB is designed to address the broad needs of tea biochemists, natural products chemists, nutritionists, and members of tea related research community.

**Conclusion:**

The TMDB database provides a solid platform for collection, standardization, and searching of compounds information found in tea. As such this database will be a comprehensive repository for tea biochemistry and tea health research community.

## Background

**Figure 1:**
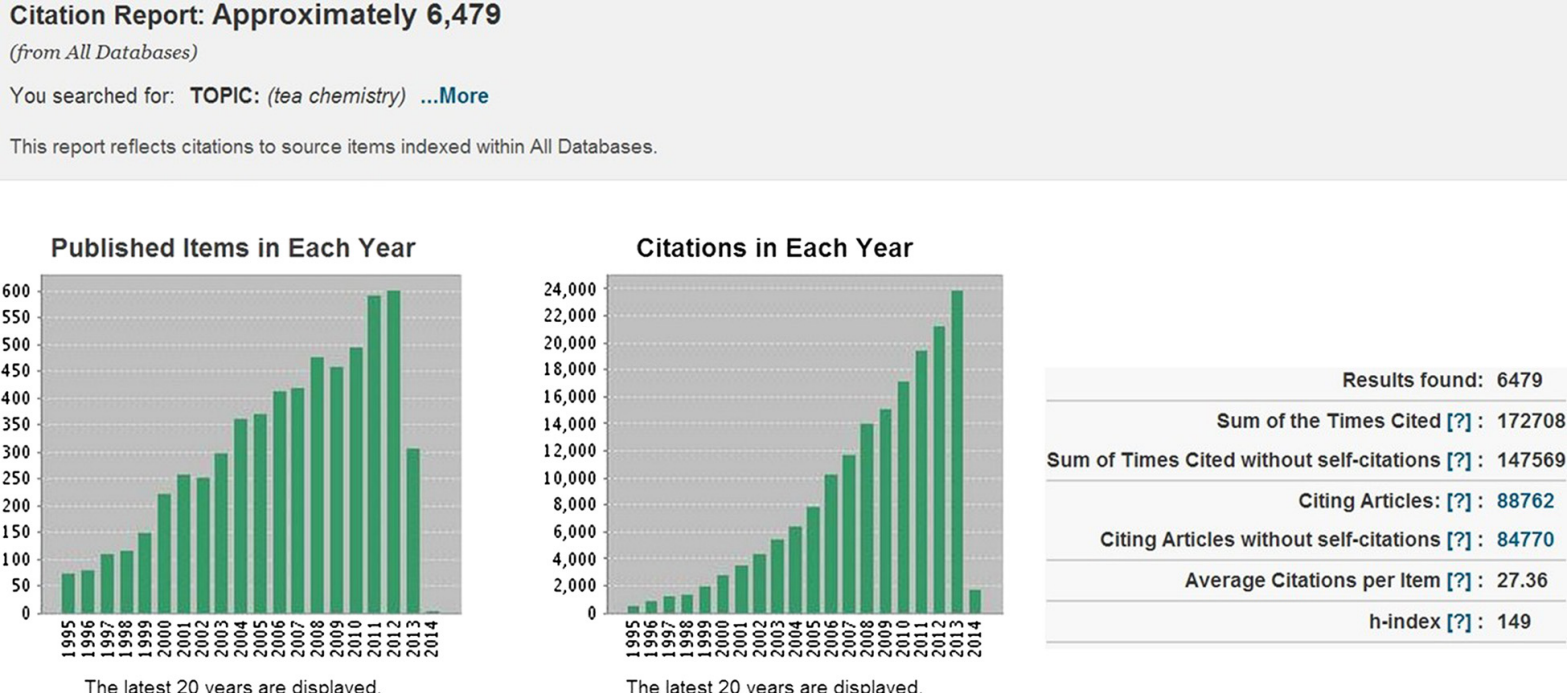
**Published items and citations for Tea chemistry during the latest 20 years.**

Tea is the second most highly consumed beverage worldwide other than water. However, unlike water, tea contains thousands of chemical components including polyphenols (mainly catechins, flavonoids and its glycosides, proanthocyanidins, phenolic acids and their derivatives), purine (xanthine) alkaloids, terpenoids and its glycosides, aroma precursors, aroma compounds, fatty acids, amino acids, carbohydrates, etc., which showed a wide spectrum of bioactivities [1-11]. Since the 1950s, tea has been vigorously researched. According to the web of knowledge, the published items on tea compounds have increased from single digit number of articles per year in 1950s to current more than 600 ones, with total citations of more than 23,000 each year now (Figure 1). The high average citations (27/item) and high h-index (149) suggested that research related to tea is a hot spot (Figure 1). However, data from these individual reports have not been collected into a single database. The lack of a curated database of related chemicals limits research in this field, and thus a cohesive database system should necessarily be constructed for data deposit and further application in related field.

Therefore, the tea metabolome project has been launched as an effort to identify all detectable compounds in tea and tea related manufactured products. In addition to experimentally identifying and quantifying hundreds of compounds in tea infusions, this multi-year project was also formally tasked with backfilling and validating the information on all previously identified compounds and providing this information as a freely available electronic database called the Tea Metabolome Database (TMDB). Most of the collecting compounds in the current TMDB version 1.0 are known compounds thanks to the work done by thousands of tea biochemists, natural products chemists, and metabolic biochemists over the last 60 years. Currently, TMDB collects 1473 compounds information, including 1393 compounds from the species *Camellia sinensis and its varieties*, 60 compounds from other *Camellia spp.*, 1 from red tea (*Leptospermum scoparium*), and 17 in vivo metabolites of tea catechins which had been found in human, mice, and rats. Flavonoids, terpenoids, purine alkaloids, and theanine are the four major types of chemicals in tea. Metabolic pathways of the four classes of compounds are now available as those in the on-line metabolic pathway database KEGG [12]. More recently, information about *C. sinensis* unigene involved the secondary metabolic pathways of flavonoids, theanine, and caffeine was also reported [13]. Although most of the compounds can be found in the already known databases, dictionaries, and textbooks such as HMDB [14-16], Dictionary of Natural Products (DNP) [17], Combined Chemical Dictironary (CCD) [18], KNApSAcK [19], Phenol-Explorer [20-22], Tea Biochemistry [23], and Grand Dictionary of Chinese Tea [24], etc., the information included in these resources does not meet the unique data requirements for tea biochemists or tea related researchers. Besides, very limited amount of chemical constituents were included in above databases (Table 1). Researchers need data directly related to chemical constituents from tea. The tea related data need to be readily available, fully referenced, easily searched and to cover as much information about tea compounds as possible. To address these needs, and to serve as a potential model for other tea metabolomic resources, we have developed the TMDB.

**Table 1:** **Different amounts of chemical constituents from the genus Camellia included in the major compounds databases or dictionaries**

**Databases**	**HMDB**	**CCD**	**DNP**	**KNApSAcK**	**TMDB**
Number of compounds from *Camellia (sinensis)*	106	312	295	250 (195)	1473 (1393)

The Tea Metabolome Database will collect the small molecular compounds (MW < 2000 Da) found in tea and its related manufactured products.

## Description, construction, content

Fundamentally, the TMDB is a multi-purpose biocheminformics database with focus on qualitative, analytic, and molecular scale description of the compounds from tea and their bioactivities. It combines enriched data from textbooks, journal articles, and other existing databases such as HMDB, Pubmed, etc. It also provides a large body of collected experimental data especially the original NMR spectra.

To compile, confirm, and validate the diverse data, the quantity and quality of experimental data, broad knowledge are needed to meet the difficult and time consuming task of constructing the TMDB. Thus, the team of TMDB includes eight tea biochemists and natural products chemists, two NMR specialists, and three bioinformticians with dual training in chemistry and computer science.

The TMDB currently contains more than 1473 compounds entries with more than 30,000 different data entry fields. The metabolic pathways for flavonoids, terpenoids, theanine, and caffeine are also provided. Hundreds of the compounds are linked to experimentally acquired or literature-sourced ^1^H, ^13^C NMR, data and the original NMR spectra as well.

## Utility

**Figure 2:**
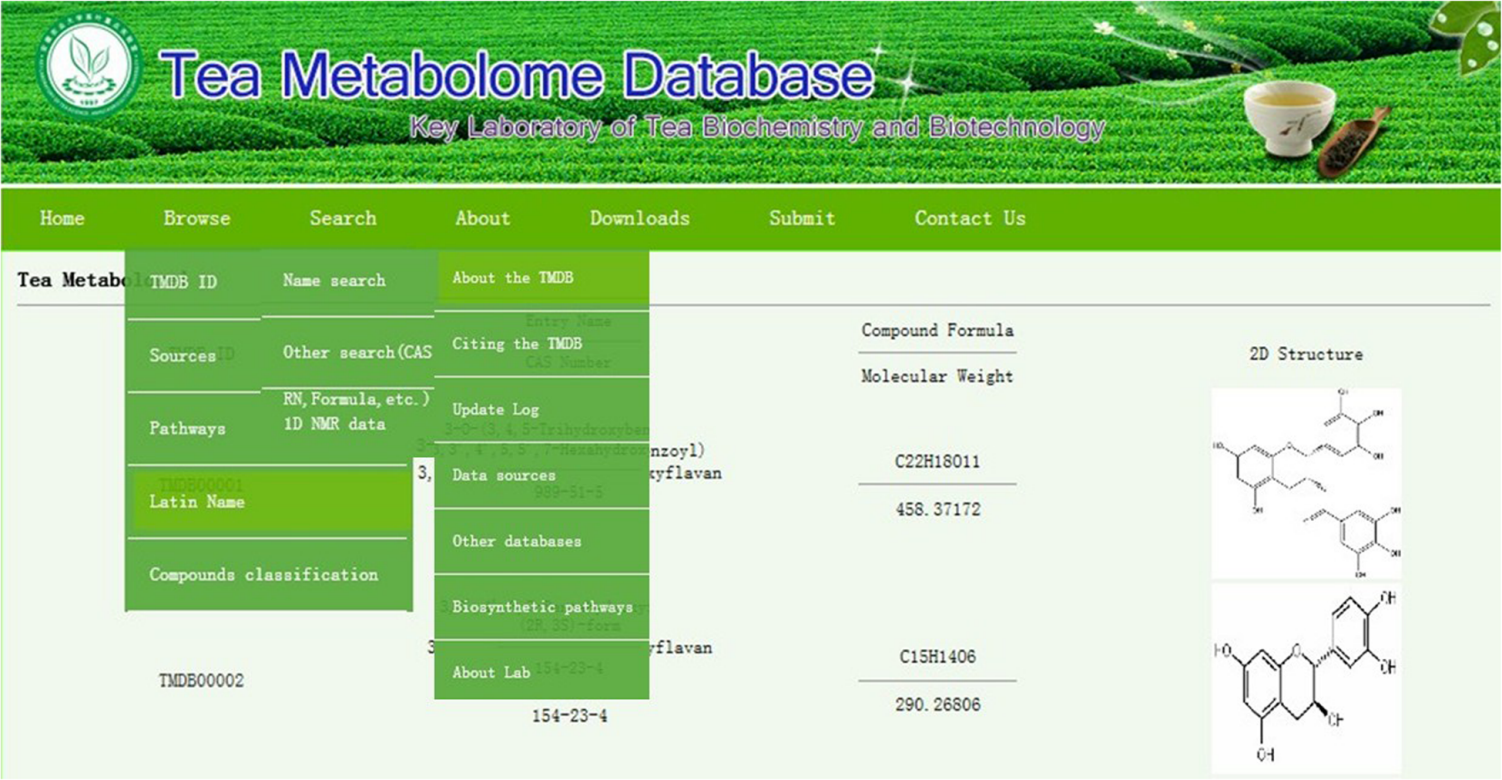
**TMDB: screenshot of drop-down box browse (left), name, other, and 1D NMR search.**

The TMDB is fully searchable with built-in tools for viewing, extracting, and sorting metabolites, NMR and bioassay data. Detailed instructions on where to locate and how to use these browsing/search tools are provided on the TMDB homepage. It supports text queries instead of specific name queries. It also offers general data browsing using the “Browse” button located in the TMDB menu bar. When you put the mouse at “Browse” button, five items (TMDB ID browse, compounds sources browse, pathway browse, Latin name browse, and compound classification browse) will appear in drop-down box (Figure 2). Clicking on the item opens a webpage describing the compound of interest in much greater detail. Each compound entry contains an average of 24 data fields (Table 2) with most of the information being devoted to chemical or physico-chemical data.

**Table 2:** **Each compound entry contains averagely 24 data fields**

**TMDB ID**	**TMDB00001**
CAS RN	989-51-5
Entry name	3-O-(3,4,5-Trihydroxybenzoyl) 3,3′,4′,5,5′,7-Hexahydroxyflavan
Synonym(s)	3-O-Galloylepigallocatechin. Teatannin II. Epigallocatechin 3-gallate. EGCG
System name	(2R,3R)-5,7-dihydroxy-2-(3,4,5-trihydroxyphenyl)chroman-3-yl 3,4,5-trihydroxybenzoate
2D structure	
Formula	C22H18O11
Mol weight	458.37172
Compound classification	Catechins, Flavonoids
Tea taxonomy	Green tea, Black tea, Oolong tea, Dark tea
Latin name	Camellia sinensis
Chemists	Bao Guanhu, Zhu Yunfei
Initial amount	1 g
Bioactivities	Shows anti-HIV activity. Tumour promotion inhibitor, putative cancer chemopreventive agent, affects tumour cell adhesion and invasion. Implicated in occup. asthma in green tea factories. Inhibits metalloproteinases
References	J. Chromatogr. A 2005,1083, 223–228; Phytochemistry 2006, 67, 1849–1855; Magn. Reson. Chem. 1996, 34, 887–890. (occur, nmr, ms)
Publication year(s)	1996. 2005. 2006
^1^H NMR data	2.71,2.92,4.86,5.42,5.87,6.42,6.86
^1^H NMR spectrum	
^13^C NMR data	25.9,68.9,77.5,94.7,95.4,98.3,105.7,109.1,120.3,129.5,132.5,138.4,144.9,145.3,155.8,156.4,156.4,166.4
^13^C NMR spectrum	
MS data	
MS/MS data	
Pathways	Flavonoid
KEGG map	map00941
HMDB ID	HMDB03153
Pubmed ID	CID 65064

**Figure 3:**
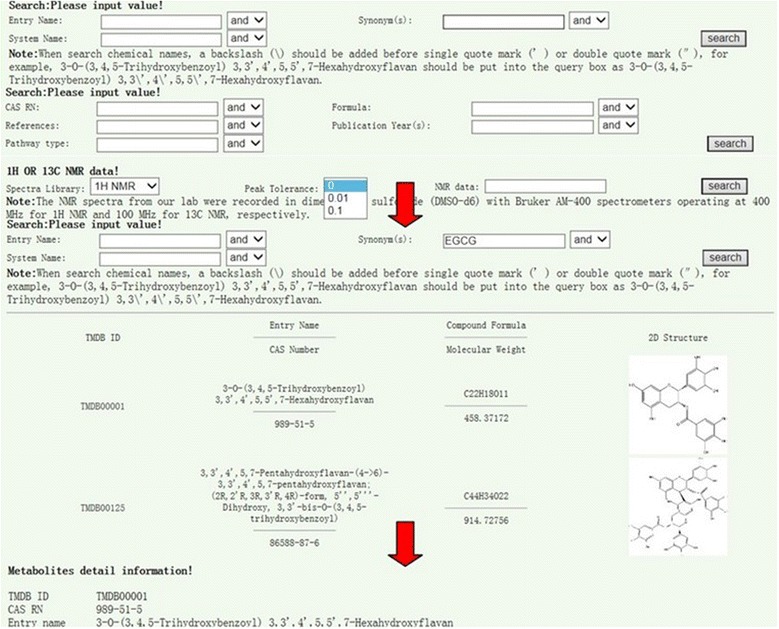
**Retrieve a compound in three search methods (name, other, and 1D NMR data), search results, and metabolites detailed information.**

It offers “Search” buttons too (Figure 2). When you put the mouse at “search” button, three search means are provided: name search, other search, and 1D NMR data search (Figure 2). Clicking on the name search, you can search the entry name, synonym, or system name of any compound you want to retrieve. For example, if you put “EGCG” in the synonym name search, several related items will appear (Figure 3). Clicking the first item, you can find metabolites detail information of the compound “EGCG”. The other search button provides CAS Registry Number, formula, references, publication year, and/or pathway type search for the compound you are interested in.

A key feature of TMDB is that it also provides searchable NMR data with the similarity identification which is highly valuable for the identification of metabolites from the crude extract and the tea metabolomics research.

### User submit to TMDB

**Figure 4:**
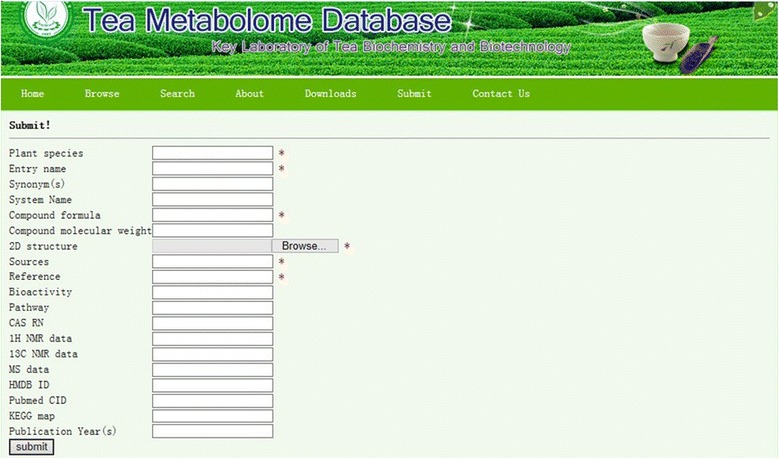
**TMDB: screenshot of the webpage from which new data can be added by an authorized compiler.**

TMDB provides a submit page that allows nonaffiliated researchers to independently contribute novel tea metabolites as they become available (Figure 4).

For user submissions, primary required fields are: 1) plant species, 2) compound name, 3) compound formula, 4) compound molecular weight, 5) compound structure, 6) NMR data, 7) MS data, 8) tea taxonomy, 9) reference. The curator(s) at our site conducts manual verification of the original publication(s) for data validation purposes to maintain the quality and integrity of the database. Submissions that pass this review process are then approved for entry into the TMDB database. Notably, all new submission will be made available in coming TMDB versions on a monthly release schedule.

### Database implementation

The TMDB is essentially a web-friendly front-end to a sophisticated MSSQL server relational database (2008 R2). Both are maintained on a Windows server 2003 equipped with a 2 GHz CPU processor and 2 GB of RAM. JSP scripts are run every night to read selected portions of the MSSQL database and to write out the data into raw text and XML formats. The raw text is dynamically rendered into HTML (with images and hyperlinks) using a series of custom JSP scripts. It is a Java application that uses Apache Tomcat server 7.0 technologies such as Java server Pages (JSP) and Java servlets to manage web-based data input and data queries.

## Discussion

**Figure 5:**
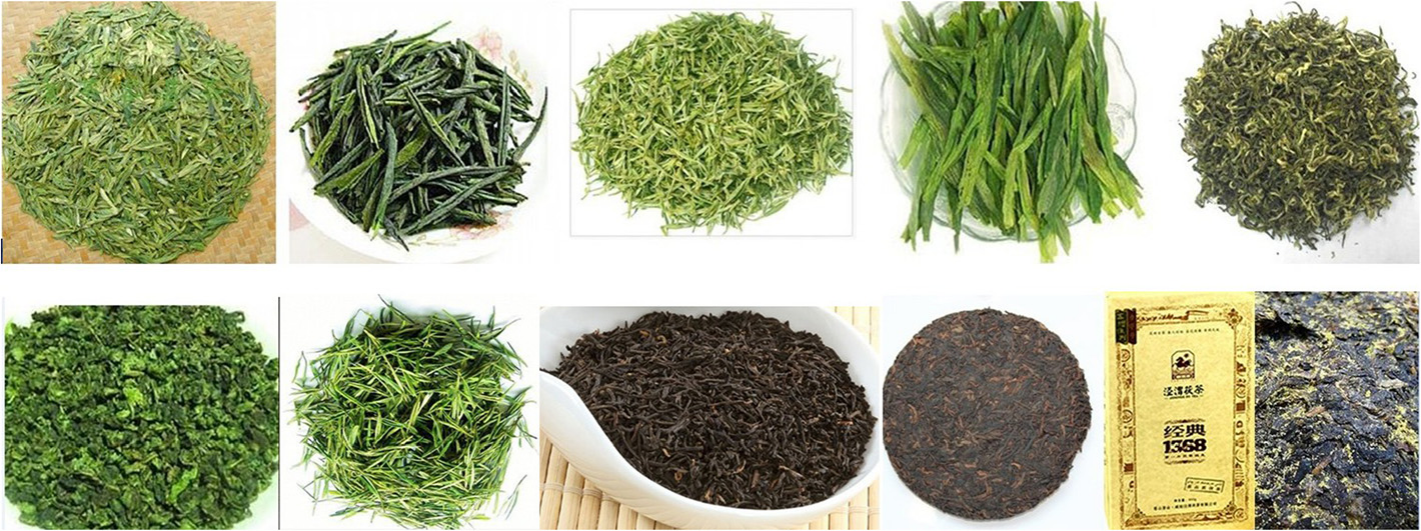
**Ten famous Chinese teas belong to the four categories of tea according to the increment of fermentation, up five: Xi-Hu Longjin, Lu-An Guapian, Huang-Shang Maofeng, Tai-Ping Houkui, Biluochun (all green tea, non-fermented); down five: Tianguanying (Oolong tea, semifermented), An-Xi white tea (partially fermented), Qi-Men also Keemun black tea (fully fermented), Pu-erh Tea (dark tea, post fermented), Jing-Wei Fu Brick tea (dark tea, post fermented).**

In China, tea related drinking has been lasted for more than three thousand of years. Along with the historical development and social progress, different kinds of tea and tea culture were developed. Up to now, tea can be classified into four major types according to the degree of fermentation: non-fermented tea (green tea including premium teas from different places of China such as Xi-Hu Longjin in Zhejiang province, Lu-An Guapian, Huang-Shang Maofeng, Tai-Ping Houkui in Anhui province, Biluochun in Jiangsu province), partially fermented tea (white tea such as An-Xi white tea from Fujian province, yellow tea such as Huo-Shang-Huang-Ya from Anhui province, and Oolong tea such as Tianguanying from Fujian province), fully fermented tea (Black tea such as the famous Qi-Men also Keemun black tea from Anhui province), and post-fermented tea (dark tea such as the famous Pu-erh tea from Yunnan province, brick tea such as Yi-Yang Fu Brick tea from Hunan province and Jing-Wei Fu Brick tea from Shanxi province, Bowl tea, and Tuo tea) [25-28] (Figure 5).

The tea metabolome database is defined as the first comprehensive collection of small molecular compounds found from tea. Constitution of the tea metabolome database depends on that a compound is found from tea or tea products including the original tea plant, the processed or manufactured tea. The molecular weight of a compound is below 2000 Da which excludes big molecules such as protein and polysaccharides. Currently, TMDB does not collect metals either. Tea compounds are first identified by literature surveys such as text books, Dictionary of Natural Products (DNP), HMDB, and most from original journal articles. Some of the data especially the NMR data and spectra are collected from experimental sources in our team (NMR experiments were carried out using a Bruker Avance 400 MHz NMR spectrometer, DMSO-*d6*, *δ* as ppm). If a compound is found to originate from tea and to be within the molecular weight limit, information about it is prepared by one member of our curation team and separately checked by other member of the team. Additional consistency checks of the information (molecular weight matches chemical formula, CAS number is correct, etc.) will be performed by senior natural products chemists. Every effort is being made to ensure the database is as complete, correct, and current as possible.

The current database collects 1473 compounds, which is almost 6–10 times the amount of other databases. Since the research history of green tea and black tea were longer, more compounds had been found in these two types of teas than others. Additionally, one compound can exist in different types of teas. 713 compounds were found in green tea and 497 were found in black tea. Oolong tea is chemically studied from 1991 and 140 compounds were reported. Although few studies have been carried out on the compounds produced from microbial fermentation process and the changing chemical components during microbial fermentation of the dark tea until the upsurge about “Pu-erh Hot”, which includes all the post-fermented teas such as Pu-erh tea, Brick tea, Bowl tea, and Tuo tea, 455 compounds are collected from these types of teas (Table 3). Interestingly, about ten B-ring fission catechins have been found specifically existing in dark tea (the post-fermented tea) [28-34], which may be produced by the microbial decomposition of tea catechins [28-35].

**Table 3:** **Compounds reported in different tea infusion and materials**

**Tea taxonomy**	**Number of compounds reported**
Green tea	713
Oolong tea	140
Black tea	497
Dark tea	455

**Figure 6:**
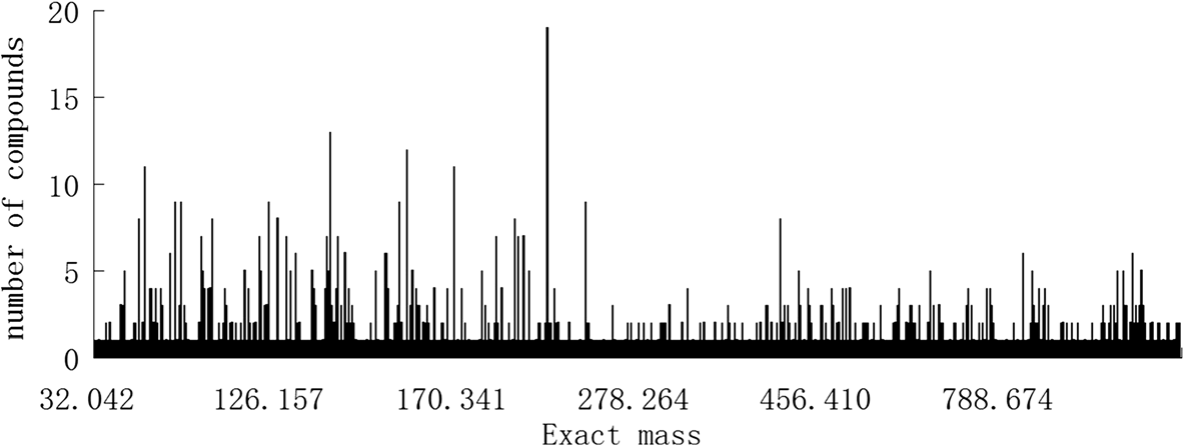
**Distribution of number of compounds per exact mass.**

Chemically, about 74% of molecular weight of the collection of compounds in TMDB is below 500 Da. More than 28% of the exact masses of the compounds correspond to a single compound which can be identified by high definition mass. However, as it can be seen from the distribution of number of compounds per exact mass in Figure 6, the amount of isomers can be up to 19, in which case the compounds of the unique chemical formulas were distributed over three or more chemical classes. This illustrates the strong need of reliable strategies for identification of these isomers which can be done to a certain extent using NMR spectra. In this case, one dimension NMR library was constructed. For NMR data queries of the 1D NMR data, EGCG as the example, the ^1^H NMR data were arranged and put in the query bar like this: 2.71,2.92,4.86,5.42,5.87,6.42,6.86 (two decimals) and the ^13^C NMR data like this: 25.9,68.9,77.5,94.7,95.4,98.3,105.7,109.1,120.3,129.5,132.5,138.4,144.9,145.3,155.8,156.4,156.4,166.4 (one decimal). Search the two series of data separately, a series of compounds will be found with the degree of similarity. Normally, the ^13^C NMR data will give better matches because of less effect of the experimental conditions on the data. Therefore, better matches can be found through ^13^C NMR data search.

The Tea metabolome database project, reported herein, provides the initial groundwork for an ongoing program for cataloging and utilizing compounds information in tea. Notably, the current version is not as comprehensive as we envision future versions to be. Ongoing collection and curation of new publications related to the concentration and MS in formation of metabolites from tea and tea products is essential to this program’s success. New versions of the databases are currently scheduled to be released on a yearly basis, incorporating all new submissions to the database over the last month of the preceding year. This practice is expected to increase comprehensiveness and accessibility of the database, promoting broader interest from researchers worldwide.

## Conclusion

We constructed the first comprehensive database for small molecular compounds from tea. The web-based program was created by manually reviewing widely scattered scientific literatures. This database, however, also provides a convenient submission interface for contribution of novel candidates by independent researchers. TMDB provides comprehensive, keyword-searchable tea compounds. Thus, TMDB will aid in rapid and complete exploration of the discovery of new compounds in tea and tea products, qualitative and quantitative analysis of tea products, standardization of the processing and manufacturing of tea and even chemical taxonomy of tea, serving as a valuable resource for future investigators in experimental chemistry and biochemistry concerned with commercial tea processing and characteristics.

## Availability and requirements

**Project name**: TMDB: A literature-curated database for small molecular compounds found from tea.

**Project home pages**: http://pcsb.ahau.edu.cn:8080/TCDB/index.jsp.

**Operating system(s)**: Platform independent.

**Programming languages**: JSP, WSSQL, HTML and JavaScript.

**License**: Not required.

**Any restrictions to use by non-academics**: None.
